# Announcing the 2022 Arnold Berliner Award Winner: spider research at the International Space Station

**DOI:** 10.1007/s00114-022-01821-w

**Published:** 2022-10-10

**Authors:** Matthias Waltert

**Affiliations:** University of Göttingen, Göttingen, Germany

**Fig. 1:**
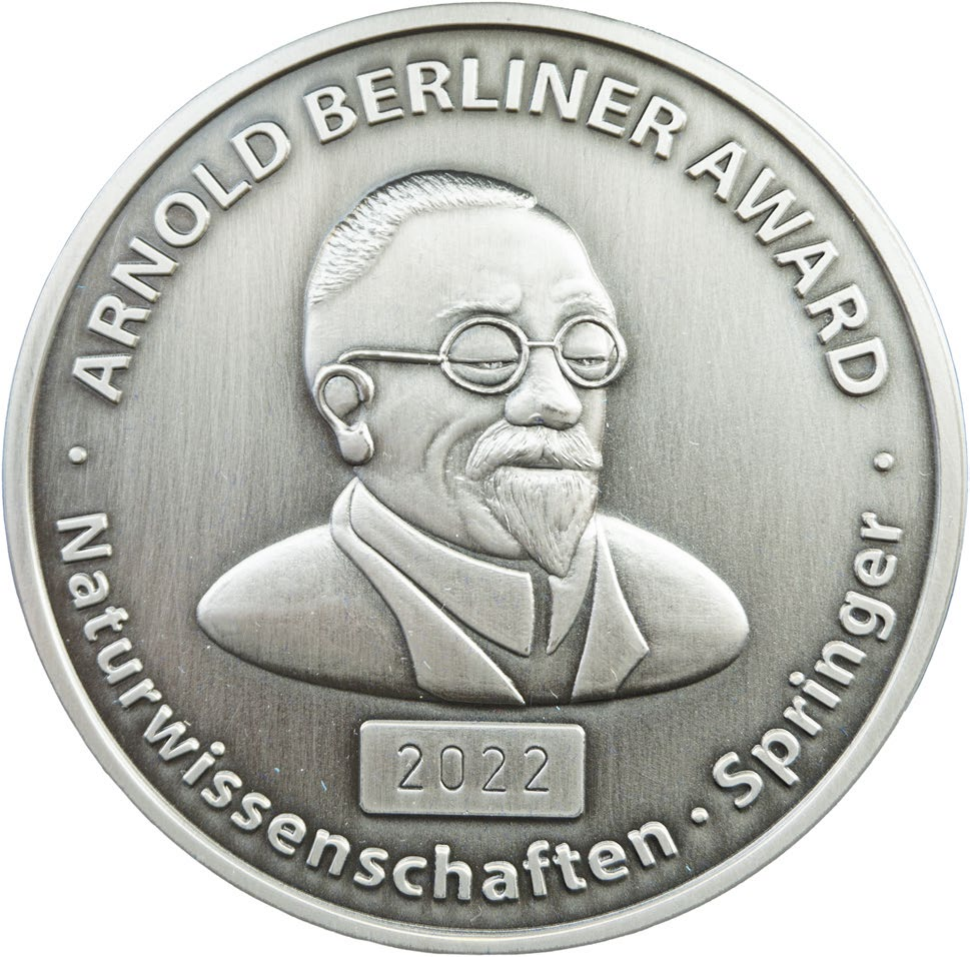
The Arnold Berliner Award medal

Every year, *The Science of Nature* grants the Arnold Berliner Award to an article’s lead author who is distinguished by excellent, original, and—especially—interdisciplinary research. As such, the winning articles clearly reflect the vision of Arnold Berliner (Autrum 1988; Thatje 2018). Springer sponsors the award which consists of four parts: the Arnold Berliner Award medal (Fig. 1), a 2-year subscription to the journal’s electronic edition, a 500-Euro voucher for Springer ebooks, and a cash prize of 250 Euro.

**Fig. 2:**
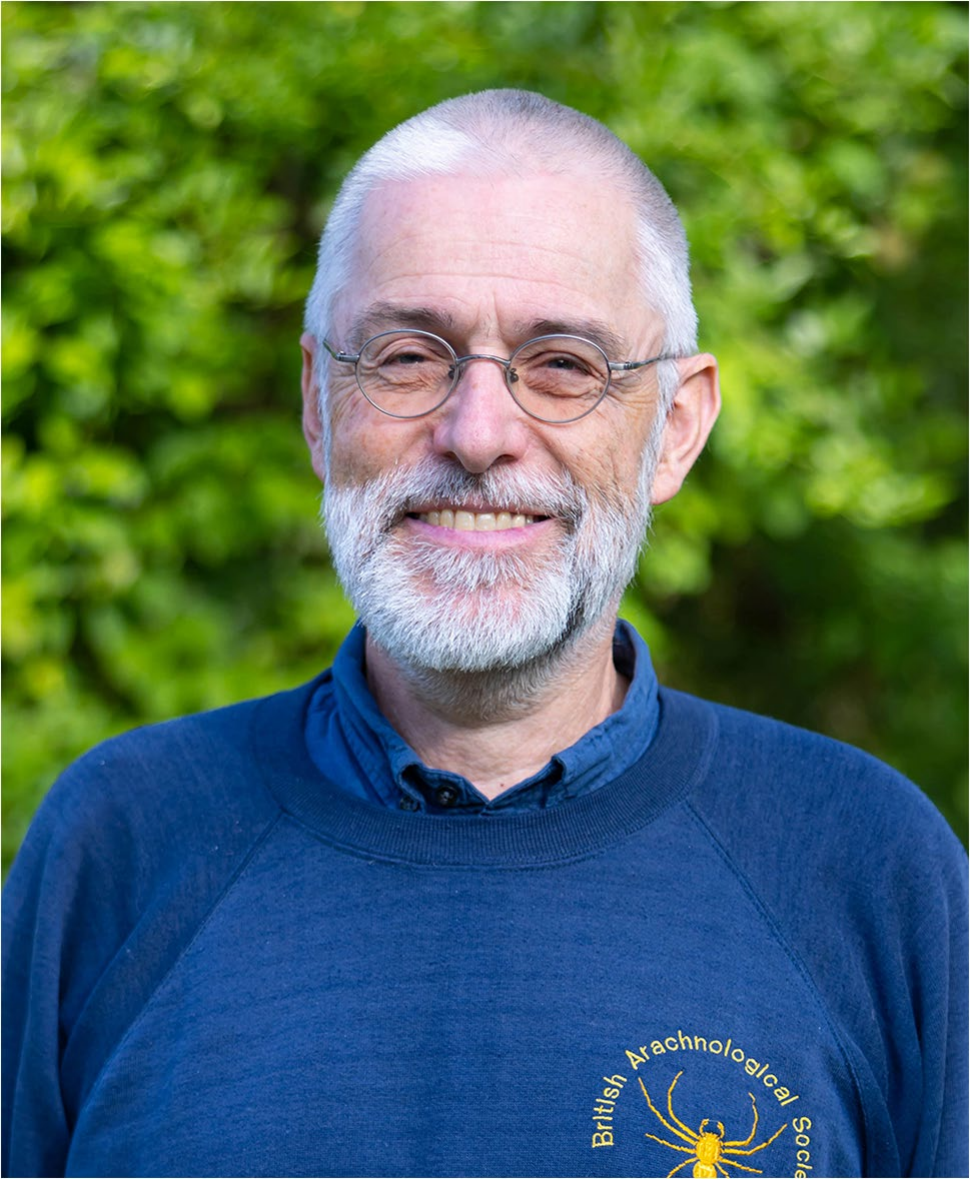
Samuel Zschokke

I am very proud that, this year, the Editorial Board has decided to award Samuel Zschokke (Fig. 2) for his article “Spiders in space—orb-web-related behaviour in zero gravity” (Zschokke et al. 2021). With their research, he and co-authors Stefanie Countryman and Paula E. Cushing demonstrate the importance of gravity for organisms. Having brought web-building spiders to the International Space Station, they observed two juvenile spiders over a 2-month period in zero gravity, and also observed two control spiders under otherwise identical conditions on Earth. Under natural conditions the studied spiders build asymmetric webs with the hub towards the upper edge of the web, and individuals orient themselves downwards when sitting on the hub whilst waiting for prey. In contrast to the natural condition, most, but not all, webs built in zero gravity were quite symmetric and closer analysis revealed that webs built when lights were on were more asymmetric (with the hub near the lights) than webs built when the lights were off. In addition, spiders showed a random orientation when the lights were off but faced away from the lights when they were on. The authors conclude that in the absence of gravity, the direction of light may serve as an orientation guide for spiders during web building and when waiting for prey on the hub.

On behalf of the Editorial Board, I congratulate Samuek Zschokke and his team on the award.
